# Biochemical parameters in the blood of Holstein calves given immunoglobulin Y-supplemented colostrums

**DOI:** 10.1186/1746-6148-10-159

**Published:** 2014-07-14

**Authors:** Teódulo Quezada-Tristán, Viridiana L García-Flor, Raúl Ortiz-Martínez, José L Arredondo-Figueroa, Leticia E Medina-Esparza, Arturo G Valdivia-Flores, Ana L Montoya-Navarrete

**Affiliations:** 1 Departamento de Clínica Veterinaria, Centro de Ciencias Agropecuarias, Universidad Autónoma de Aguascalientes, Aguascalientes, México; 2 Instituto Tecnológico El Llano Aguascalientes, Aguascalientes, México

**Keywords:** Calves, Colostrum, GGT, IgG, IgY, TP

## Abstract

**Background:**

In any calf rearing system it is desirable to obtain healthy animals, and reduce morbidity, mortality, and economic losses. Bovine syndesmochorial placentation prevents the direct transfer of bovine immunoglobulins to the fetus, and calves are born hypogammaglobulinemic. These calves therefore require colostrum immediately after birth. Colostrum is rich in immunoglobulins (Ig) and its consumption results in the transfer of passive immunity to calves. The Ig absorption occurs within the first 12 h after birth. Immunoglobulin Y (IgY), derived from chicken egg yolk, has been used in the prevention and control of diseases affecting calves because it is very similar in structure and function to immunoglobulin G (IgG). In the current study, we sought to establish whether administration routes of colostrum supplemented with avian IgY affected passive immunity in calves.

**Results:**

No significant differences were observed with respect to route of administration for colostrum. However, we did observe some differences in certain interactions between the various treatments. Calves fed colostrum containing egg yolk had higher levels of TP, ALB, and IgG, along with increased GGT activity.

**Conclusions:**

Our results suggest that supplementing colostrum with egg yolk has a beneficial effect when given to calves, regardless of administration route.

## Background

During calf rearing it is desirable to reduce morbidity and mortality, and to lower costs by avoiding expensive treatments and losses that are a result of late development and delayed production. To achieve these goals, it is necessary to ensure an adequate intake of colostrum to calves during the neonatal period, thereby providing passive immunity [1]. The most important factor in the development of calves is the appropriate and immediate consumption of colostrum post-partum, as it is the first source of nutrients after birth [2]. This should not be delayed for more than 9 h after birth. For the adequate transfer of passive immunity *via* colostrum, different feeding methodologies have been developed that vary in complexity, accessibility and cost. The transfer of passive immunity is based on different components of colostrum that are absorbed by the gastrointestinal tract of calves [3]. At the end of gestation the mammary gland of the cow produces colostrum, achieving maximum production in the last weeks of pregnancy. Colostrum is an important source of antibodies (Abs) and its absorption is essential in protecting calves against enteric infections, the main cause of death during the first weeks of life [4].

The immunological characteristics of colostrum are high for 4 days after delivery. However, its most potent immunological qualities are lost at 14 h post-partum [5] because immunoglobulins (Ig) concentrations progressively decrease [4]. The number of pregnancies for a cow has a remarkable impact on the volume and quality of produced colostrum. In multiparous cows, colostrum is richer in Abs, thus providing better immunity to calves. Another factor affecting colostrum quality is the handling of the dry cow period, where adequate nutrition and rest between drying off and calving must be ensured [6]. Other factors such as udder conformation, teat size, maternal instinct and dystocia have been associated with a failure to transfer passive immunity in calves [7,5].

The function of active Abs in the immune system is to neutralize and opsonize bacteria and other foreign particles invading an organism [8]. The concentration of Igs in cow colostrum ranges 50–150 mg/mL [9] and is composed of immunoglobulin G (IgG), immunoglobulin A (IgA) and immunoglobulin M (IgM). Two subclasses of IgG, IgG1 and IgG2, comprise 80–85% of all colostrum Igs, while IgA comprises 8–10% and IgM 5–12%. These Ig molecules provide immunity against a wide variety of systemic infections and diseases in cattle [10].

Colostrum is the only food source that transfers passive immunity until a calf develops its own active immunity, which takes at least 6 weeks [11]. The absorption of intact Ig molecules occurs for the first 12 h after birth, after which intestinal tract absorption decreases significantly until 72 h after birth, when no Igs are absorbed [4]. Kaske et al. [2] reported the existence of significant changes in Ig absorption that were dependent upon the way colostrum was fed to calves.

Antibodies are employed in various roles in biomedical studies; they are usually obtained from mammals [12]. However, in recent years, chicken IgY has been increasingly used [13] as it can be easily extracted from egg yolks. In addition to aspects related to animal welfare, the levels of Abs produced by chickens are greater than those obtained from various animals, in particular rabbits [14]. From an economic point of view, the use of IgY has a unique advantage. The cost of raising a chicken is no different than that of a rabbit. A significant amount of IgY can be produced from a single hen, between 17–35 g/bird/year. The relatively low cost IgY production allows it to be applied to immunotherapy and immunoprophylaxis of viral and bacterial infections in human and veterinary medicine [12]. Following extraction and purification from egg yolk, the concentration of IgY ranges 100–400 mg/egg yolk, with an average yolk volume of 15 mL [11,15,16]. Variations in the concentrations of IgY are dependent upon chicken strain or breed, and genetics [17-20]. IgY from chicken egg yolk is an important alternative that could help improve the immune system of Holstein calves.

In our study, we sought to establish whether different routes of colostrum administration, and supplementation of colostrum with chicken IgY affected passive immunity during calf rearing.

## Methods

### Animal study

The study was conducted at the “Las Jarillas” ranch facilities in Aguascalientes City, Aguascalientes, Mexico. The Animal Care Committee of Universidad Autónoma de Aguascalientes authorized our study in compliance with the Guide for Care and Use of Laboratory Animals [21]. We selected 30 female calves with the following characteristics: not born from dystocia; without signs of congenital or acquired problems; and no colostrum intake. All calves had an average weight of 38.0 ± 3.0 kg, and did not present with signs of diseases. We used randomized blocks with a factorial arrangement (2 × 3 × 6), resulting in 36 treatments. An esophageal tube or bottle was used to administer colostrum. The amount of egg yolk used to supplement colostrum was 0, 150, and 300 g, corresponding to 0, 1200, and 2400 mg of IgY, respectively. We sampled blood from calves at six intervals (2, 12, 24, 72, 120, and 168 hours).

There were six regimens that we conducted, with each repeated five times. Treatments 1–3 involved colostrum fed by bottle supplemented with 0, 150 and 300 g of egg yolk, respectively. Treatments 4–6 involved colostrum administered *via* an esophageal tube supplemented with 0, 150 and 300 g of egg yolk, respectively.

Calves were weighed and measured immediately after birth and then randomly allocated to one of the six treatment groups. Animals were house in a single hutch with a soil floor that was previously disinfected, dried, and roofed. Buckets for water and food were provided. All calves were fed within the first 2 h after birth with colostrum from their own dam; the amount of colostrum given was 10% of their body weight. We obtained 2610 eggs from a single batch of Hy Line W-36 hens (60 weeks old; average weight, 62.0 ± 3.0 g). The yolks from these eggs were used to obtain IgY with the aid of an IgY Eggs Press Purification Kit (Gallus Immunotech Inc., Canada). Yolks were separated from eggs, and pooled to provide 150 g and 300 g egg yolk preparations, placed in plastic bags and diluted 1:1 with tap water, and then refrigerated until required. Egg yolk preparations were administered at 2, 12, 24, and 72 h post-partum for the respective treatment groups. We obtained blood samples (5 mL) from calves by jugular venipuncture at 2, 12, 24, 72, 120 and 168 h post-partum. Blood samples were centrifuged (3000 rpm, 10 min) and the resulting serum was stored at -20°C until analysis.

### Determination of biochemical parameters in blood samples

To determine aspartate transferase (AST), alanine transferase (ALT), and gamma-glutamyl transferase (GGT) activities we used the AST/GOT Spinreact™, ALT/GPT Spinreact and GGT Spinreact kits, respectively, from Spinreact (Girona, Spain). Samples for AST and ALT determination were analyzed by spectrophotometry using an RA-5O Chemistry Analyzer (Bayer) with a wavelength of 340 nm, as described by Murray and Kaplan [22]. Samples for GGT determination were analyzed using the spectrophotometric method described by Genders and Kaplan [23]. Total protein (TP) concentration was determined using a TP Spinreact kit (Spinreat) and the method described by Burthis and Ashwood [24]. The concentration of albumin (ALB) in samples was measured using the spectrophotometric method described by Genders and Kaplan [23] in conjunction with a the ALBUMIN Spinreact kit (Spinreact). We measured the concentrations of IgG in samples using a technique described by Kliks et al. [25].

### Statistical analysis

Our data were analyzed through analysis of variance (ANOVA) with a general lineal model (GLM) and Statistical Analysis System (SAS) software [26]. A multiple range of means was compared using Tukey’s test. A *P*-value less than 0.05 was considered statistically significant.

## Results

**Figure 1:**
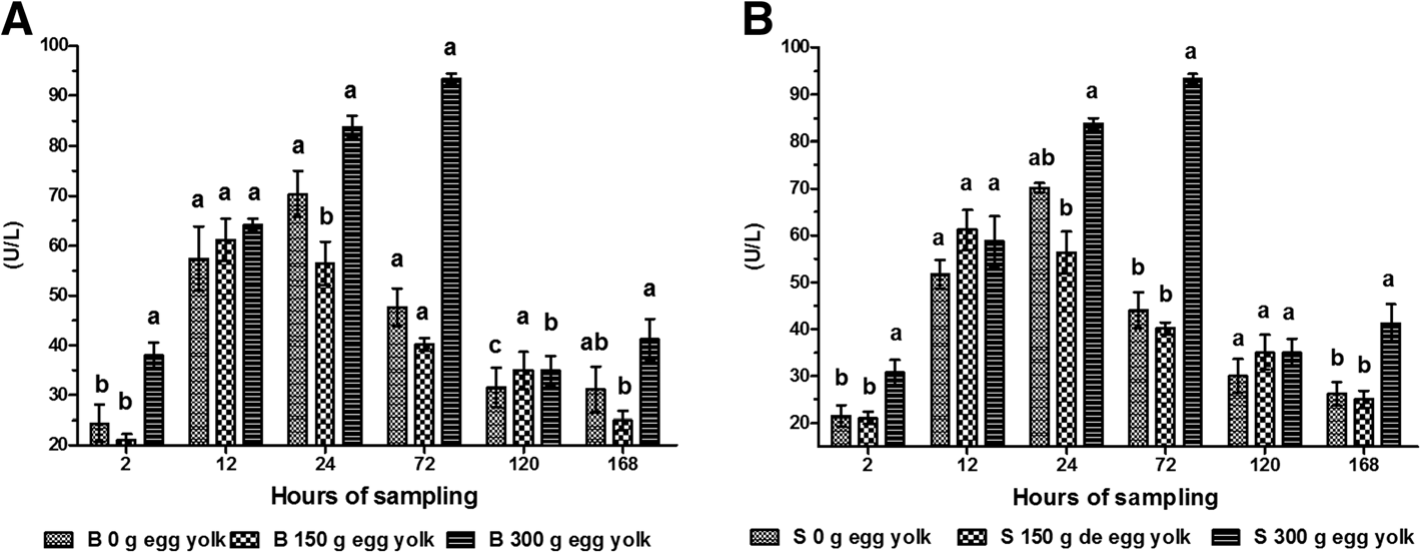
**Serum activity of AST in calves.** Calves were fed colostrum *via* bottle **(A)** or esophageal tube **(B)**. Blood sampling occurred at 2, 12, 24, 72, 120 and 168 h post-partum. Colostrum was supplemented with tree levels of egg yolk (0, 150 y 300 g) corresponding to 0, 1200 and 2400 mg of IgY respectively. The various letters represent statistically significant difference (*P* < 0.05).

**Figure 2:**
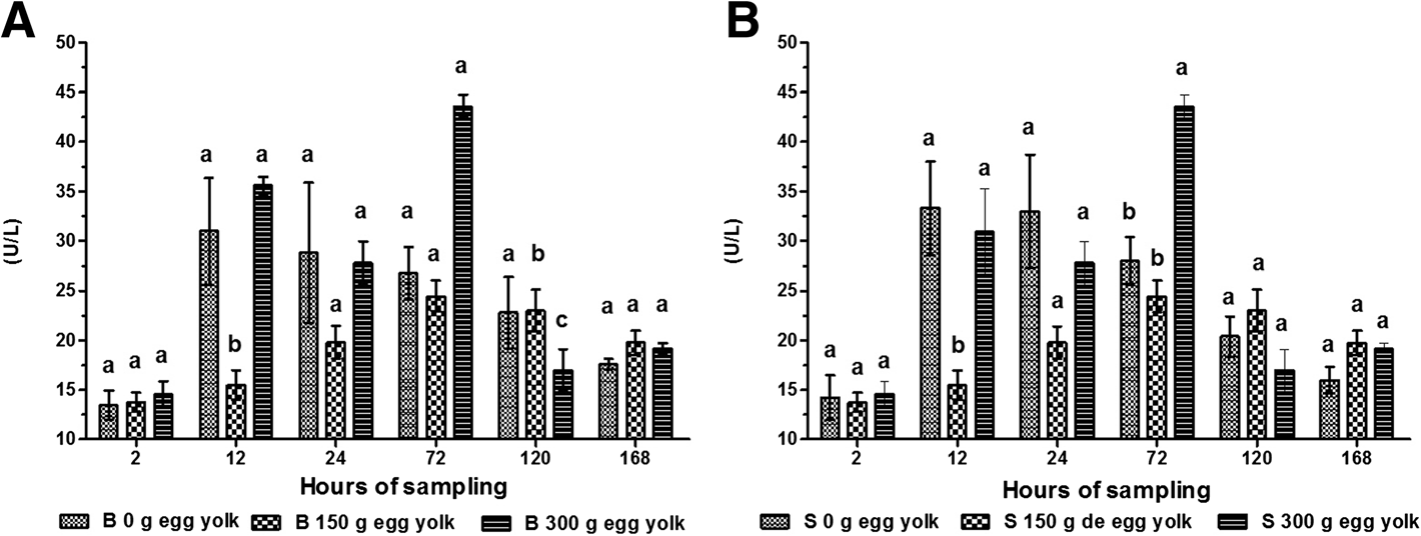
**Serum activity of ALT in calves.** Calves were fed colostrum *via* bottle **(A)** or esophageal tube **(B)**. Blood sampling occurred at 2, 12, 24, 72, 120 and 168 h post-partum. Colostrum was supplemented with tree levels of egg yolk (0, 150 y 300 g) corresponding to 0, 1200 and 2400 mg of IgY respectively. The various letters represent statistically significant difference (*P* < 0.05).

The dosage, time and interaction for all variables were highly significant (*P* < 0.001), as were the interactions between feeding method and dose, and feeding method and time for IgG (Table 1).The activity of AST in calves given 300 g of egg yolk was highest after 72 h irrespective of administration route (Figure 1A and B). AST activity was lowest when 150 g of egg yolk was administered at 2 h post-partum (bottle fed: 21.10 ± 1.20 U/L; tube fed: 21.00 ± 1.18 U/L). ALT activity was highest (bottle fed: 43.60 ± 1.16 U/L; tube fed: 43.50 ± 1.14 U/L) at 72 h post-partum. ALT activity was lowest in calves given 150 g of egg yolk at 2 h post-partum (bottle fed: 13.75 ± 0.97 U/L; 13.45 ± 1.50 U/L) and in calves that were not given any egg yolk. For both these groups, from 72–168 h after birth, there was a continual decrease in ALT activity (Figure 2A and B).

**Table 1:** Analysis of variance for certain biochemical parameters in the blood of newborn calves

**Resource**	**DF**	**AST**	**ALT**	**GGT**	**PT**	**ALB**	**IGG**
		**Pr > F**	**Pr > F**	**Pr > F**	**Pr > F**	**Pr > F**	**Pr > F**
**M**	1	0.1994 **ns**	0.9949 **ns**	0.3335 **ns**	0.8802 **ns**	0.7220 **ns**	0.3636 **ns**
**D**	2	<0.0001***	<0.0001***	<0.0001***	<0.0001***	<0.0001***	<0.0001***
**M × D**	2	0.4306 **ns**	0.7771 **ns**	0.7414 **ns**	0.8875 **ns**	0.6599 **ns**	0.7858 **ns**
**T**	5	<0.0001***	<0.0001***	<0.0001***	<0.0001***	<0.0001***	<0.0001***
**M × T**	5	0.9718 **ns**	0.9758 **ns**	0.6860 **ns**	0.9988 **ns**	0.9994 **ns**	0.8011 **ns**
**D × T**	10	<0.0001***	<0.0001***	<0.0001***	0.0331 **ns**	0.0031 **ns**	<0.0001***
**M × D × T**	10	1.0000 **ns**	0.9873 **ns**	0.8194 **ns**	0.9995 **ns**	0.9996 **ns**	0.5043**

M, method; D, dose, M × D, intersection method and dose; T, time; M × T, intersection of method and time; D × T, intersection of dose and time; M × D × T, intersection method, dose and time; AST, aspartate transferase; ALT, alanine transferase; GGT, gamma glutamyl transferase; TP, total protein; ALB, albumin; IGG, immunoglobulin G; ns, no significant difference; ^**^significant difference; ^***^highly significant difference.

**Figure 3:**
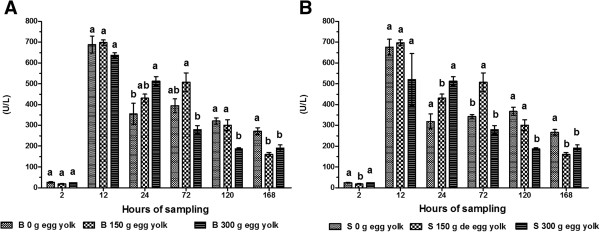
**Serum activity of GGT in calves.** Calves were fed colostrum *via* bottle **(A)** or esophageal tube **(B)**. Blood sampling occurred at 2, 12, 24, 72, 120 and 168 h post-partum. Colostrum was supplemented with tree levels of egg yolk (0, 150 y 300 g) corresponding to 0, 1200 and 2400 mg of IgY respectively. The various letters represent statistically significant difference (*P* < 0.05).

**Figure 4:**
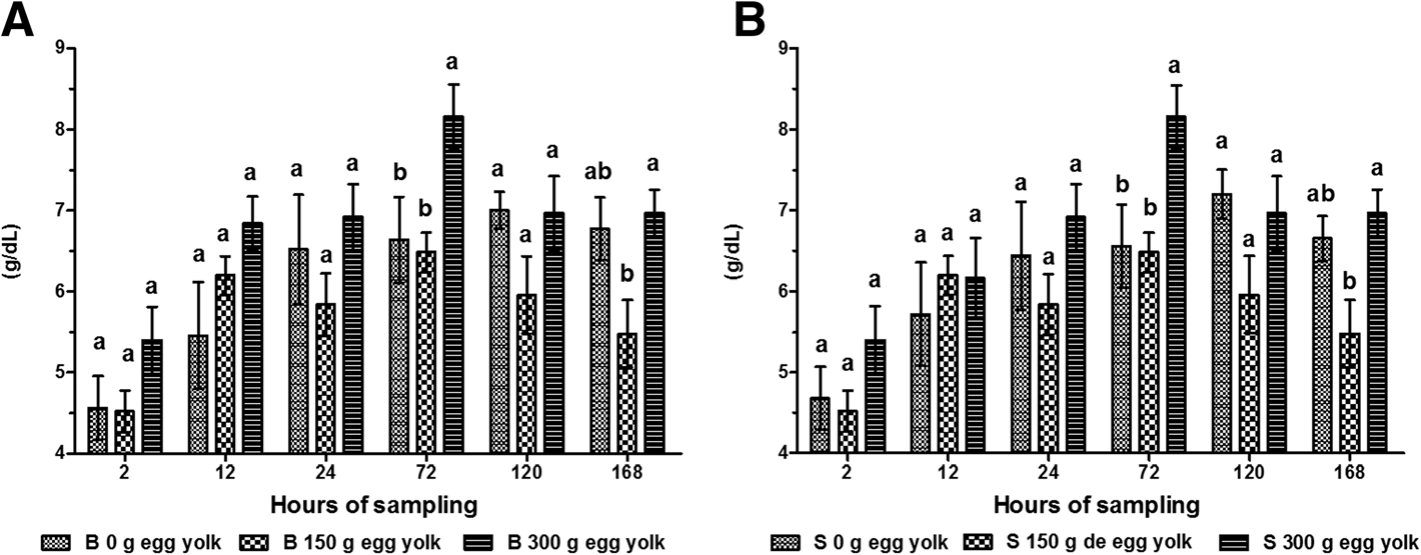
**Serum TP concentration in calves.** Calves were fed colostrum *via* bottle **(A)** or esophageal tube **(B)**. Blood sampling occurred at 2, 12, 24, 72, 120 and 168 h post-partum. Colostrum was supplemented with tree levels of egg yolk (0, 150 y 300 g) corresponding to 0, 1200 and 2400 mg of IgY respectively. The various letters represent statistically significant difference (*P* < 0.05).

We did not observe a significant difference in GGT activity between calves receiving colostrum through a tube or a bottle. However, we did observe a significant difference between treatments. GGT activity was highest in calves that received 150 g of egg yolk regardless of administration route (bottle fed: 697.80 ± 13.30 U/L; tube fed: 696.5 ± 13.0 U/L). The GGT activity in animals given 300 g of egg yolk at 12 and 24 h was 637.00 ± 12.61 and 520.6 ± 12.5 U/L, respectively (*P* < 0.01). GGT activity was lowest at 2 h post-partum for both routes of administration (bottle fed: 18.6 ± 1.2 U/L; tube fed: 18.6 ± 1.18 U/L; *P* < 0.05). The activity of GGT gradually decreased from 24–168 h post-partum (Figure 3A and B).We observed an increase in TP concentration 12 h after the consumption of colostrum in all treatment groups (Figure 4A and B). This increase was more pronounced in calves that received 300 g of egg yolk by both administration routes (bottle fed: 8.16 ± 0.38 g/dL; tube fed: 8.17 ± 0.39 g/dL). The concentration of TP was lowest in calves at 2 h post-partum (bottle fed: 4.52 ± 0.25 g/dL; tube fed: 4.53 ± 0.26 g/dL). There was a gradual decrease in TP concentration from 72 to 168 h.

**Figure 5:**
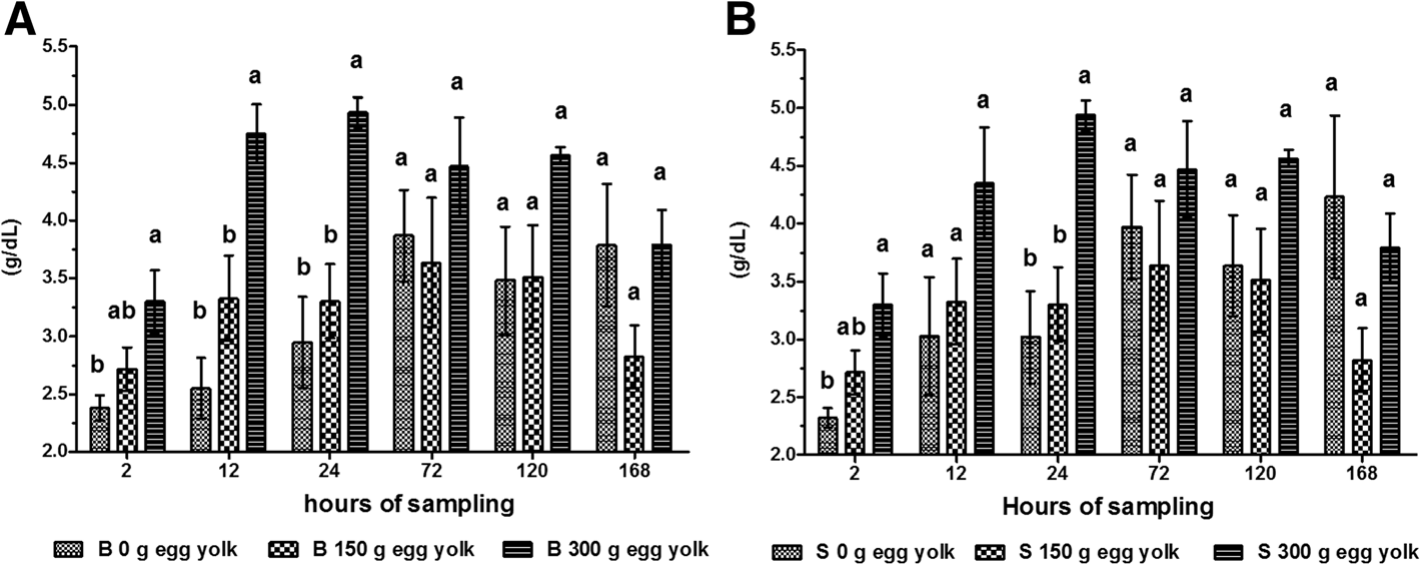
**Average serum ALB concentration in calves.** Calves were fed colostrum *via* bottle **(A)** or esophageal tube **(B)**. Blood sampling occurred at 2, 12, 24, 72, 120 and 168 h post-partum. Colostrum was supplemented with tree levels of egg yolk (0, 150 y 300 g) corresponding to 0, 1200 and 2400 mg of IgY respectively. The various letters represent statistically significant difference (*P* < 0.05).

The concentration of ALB was highest in calves that received 300 g of egg yolk for both feeding methods (bottle fed: 4.93 ± 0.13 g/dL; tube fed: 4.94 ± 0.12 g/dL) 24 h after birth (*P* < 0.01; Figure 5A and B). The concentration of ALB was lowest in calves administered colostrum 2 h after birth and where egg yolk was not a supplement (bottle fed: 2.38 ± 0.11 g/dL; tube fed: 2.32 ± 0.08 g/dL; *P* < 0.05). We also observed a decrease in ALB levels between 72–168 h that was independent of colostrum administration route; this was only seen in treatment groups were egg yolks were excluded from colostrum (*P* < 0.01).

**Figure 6:**
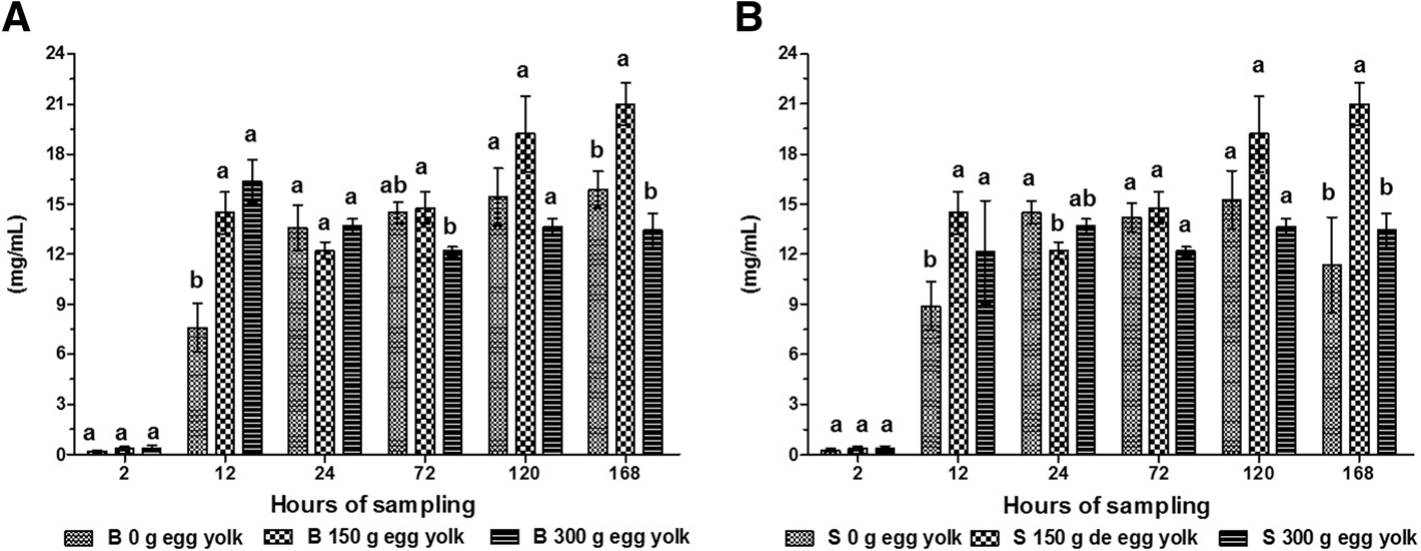
**Average serum IgG concentration in calves.** Calves were fed colostrum *via* bottle **(A)** or esophageal tube **(B)**. Blood sampling occurred at 2, 12, 24, 72, 120 and 168 h post-partum. Colostrum was supplemented with tree levels of egg yolk (0, 150 y 300 g) corresponding to 0, 1200 and 2400 mg of IgY respectively. The various letters represent statistically significant difference (*P* < 0.05).

The highest IgG concentrations were observed 12 h after birth in calves that received 150 or 300 g of egg yolk and were administered colostrum by bottle (14.48 ± 1.26 mg/mL and 16.36 ± 1.28 mg/mL, respectively; *P* < 0.05; Figure 6A and B). Lower IgG concentrations were seen in the control groups fed with a bottle (0.20 ± 0.07 mg/mL) and in tube-fed calves 2 h after birth (0.029 ± 0.08 mg/mL). The concentration of IgG in samples did not significantly fluctuate for the duration of the experimental period (168 h). Calves fed colostrum supplemented with 150 g of egg yolk exhibited a gradual increase in IgG concentration from 72 to 168 h (*P* < 0.05).

## Discussion

The measured activities of AST and ALT in blood samples were highest at 72 h and could be considered within the normal reference range. These high levels indicate that the corresponding enzymes were present in the colostrum of newly calved cows. Once AST and ALT, along with other enzymes, are ingested *via* colostrum they are absorbed by the calf intestine before secretion into the bloodstream. The activities of hepatic AST and ALT are increased during pathological processes associated with necrosis. Following this increase in hepatic activity, these enzymes are released into the bloodstream [27]. These two enzymes are used in tests to diagnose liver damage [24,28]. An AST value between 5–40 U/L is considered normal [29], while an ALT value ranging 5–150 U/L is considered normal [30]. In the current study, our results fall within the normal range. We used the activity of these enzymes to verify the increase in GGT activity in calf sera.

The increase in GGT activity at 12–24 h post-partum occurs because the mammary gland is at rest, preparing for the next lactation event, and producing and storing large quantities of colostrum rich in antibodies. GGT is a membrane enzyme found at significantly high concentrations in the cells of alveolar tissue discharged into colostrum. GGT is easily absorbed by newborn ruminants, and serum GGT activity is high in calves that have consumed colostrum [31]. Others have reported 16-fold increases in GGT activity for colostrum-fed calves at 24 h after birth [32]. A correlation between consumption of colostrum and GGT activity has been shown [33]. Absorption of GGT and Ig in colostrum is limited to the first 24 h after birth. In newborn calves, GGT activity is around 0 U/L but then increases to 1773 U/L at 24 h post-partum following the consumption of colostrum [34]. Our results in the current study support these previous findings, suggesting that serum GGT levels in calves can be used as a qualitative indicator for adequate transfer of passive immunity.

The TP concentration for calves administered colostrum by either route increased by 80.5% at 24 h post-partum. Increases in TP concentration are dependent on the quantity and quality of colostrum provided to calves in the first 2 h after birth. TP is absorbed by the intestine of newborn calves between 12–24 h post-partum. TP concentration can be used diagnostically to reflect the immunity of a calf [3]. A concentration of TP in serum lower than 60 g/L indicates a deficiency in passive immunity as is known as failure transfer of passive immunity (FTPI). Studies have shown that the average TP concentration in calves at birth was 43 g/L, increasing to 74 g/L after 24 h [34]. Other studies have reported the serum TP concentration in calves at 72 h post-partum was 60 g/L [35]. Our results are similar to those previously seen in newborn calves (2 h post-partum). However, the concentration of TP at 24 and 72 h post-partum that we determined was 15% higher than those reported by others. Our results show that egg yolks significantly increased TP values and suggest there is a positive correlation between TP and IgG concentrations.

The concentrations of ALB in calves that received the equivalent of 2400 mg of egg yolk in colostrum were 49.3 ± 1.3 and 49.4 ± 1.2 g/L for bottle- and tube-fed animals, respectively, at 24 h post-partum (*P* < 0.05). In colostrum, similar to what occurs in serum, ALB functions as a transport protein; it is absorbed through the intestine of calves and its levels decrease 3 days after birth [36]. The concentrations of ALB and TP reflect the immunity of calves and can be used diagnostically. The concentrations of ALB we determined in the current study were higher than those previously reported [37-39]. Our results suggest that supplementing colostrum with egg yolk influences ALB concentration in the serum of calves.

The concentration of IgG was 16.36 ± 1.28 mg/mL in calves bottle-fed with colostrum containing 300 g of egg yolk at 12 h post-partum. The beneficial effects of Ig in colostrum are generally conferred within the first 2 h of life. These are well absorbed by the intestine before they are discharged into the blood at optimal concentrations. This results in an improved immune status for calves and they become resistant to farm pathogens. It was previously reported that colostrum contains 50–100 g/L IgG [35]. Other researchers have reported a positive correlation between serum IgG and TP concentrations [40,41]. In one study, the plasma IgG concentration in calves 3–8-days-old was less than 10.0 g/L [42]. In another study, plasma IgG levels ranged 3.5–47.0 mg/mL [43]. Our results in the current study indicate higher concentrations of IgG in serum, which conflicts with previous studies. A strong positive correlation between IgG, IgA, IgM and total serum protein concentrations has been demonstrated in newborn calves [44]. In our study, we found a positive correlation between the concentration of either TP or ALB and IgG.

## Conclusions

Our findings in this study indicate that egg yolk has a beneficial effect on Holstein calves when administered together with colostrum.

## Abbreviations

Abs: Antibodies; ALT: Alanine transferase; ALB: Albumin; ANOVA: Analysis of variance; AST: Aspartate transferase; GLM: General lineal model; FTPI: Failure transfer of passive immunity; GGT: Gamma-glutamyl transferase; Ig: Immunoglobulins; IgA: Immunoglobulin A; IgG: Immunoglobulin G; IgM: Immunoglobulin M; IgY: Immunoglobulin Y; SAS: Statistical analysis system; TP: Total protein.

## Competing interests

The authors declare that they have no competing interests.

## Authors’ contributions

TQT research design, experimental design; VLGF conduct research; ROM instrument and technical advice; LEME conduct experiments; AGVF and JLAF statistical and quantitative analysis; ALMN analytical determinations. All authors prepared and approved the final manuscript.

## References

[B1] Gabriel SD Duchateua LP Chembensofu M Vercruysse J The influence of colostrum on infection of calves around 7 months of ages with Schistosoma mattheei Vet Parasitol 2005 129 55 60 15817203 10.1016/j.vetpar.2004.11.034

[B2] Kaske MW Schuberth HJ Rehage JK Colostrum management in calves: effects of drenching vs. bottle feeding J Anim Physiol Anim Nutr 2005 89 151 157 10.1111/j.1439-0396.2005.00535.x 15787987

[B3] Gungor O Bastan A Erbil MK The usefulness of the-glutamyltransferase activity and total proteinemia in serum for detection of the failure of immune passive transfer in neonatal calves Revue Med Vet 2004 155 27 30

[B4] Campos R Fairut AC Loaiza V Leonidas G Colostrum: Tool for rearing calves 2007 National University of Colombia: Headquarters Palmira, Department of Science Animal

[B5] Moore M Tyler JW Chigerwe M Dawes ME Middleton JR Effect of delayed colostrum collection on colostral IgG concentration in dairy cows J Am Vet Med Assoc 2005 226 1375 1377 15844432 10.2460/javma.2005.226.1375

[B6] Filteau VE Bouchard G Fecteau L Dutil DD Health status and risk factors Associated with failure of passive transfer of immunity in newborn beef calves in Quebec Can Vet J 2003 44 907 913 14664353 PMC385448

[B7] McGurk SM Collins M Managing the production, storage, and delivery of colostrum Vet Clin N Am-Food A 2004 20 593 603 10.1016/j.cvfa.2004.06.005 15471626

[B8] Maunsell FP Morin DE Constable PD Hurley WL McCoy GC Use of mammary gland and colostral Characteristics for prediction of colostral IgG1 concentration and intramammary infection in Holstein cows J Am Vet Med Assoc 1999 214 1817 1822 10382025

[B9] Foster DM Smith GW Sanner TR Busso GV Serum IgG and total protein concentrations in dairy calves fed two colostrum replacement products J Am Vet Med Assoc 2006 229 1281 1285 10.2460/javma.229.8.1282 17042734

[B10] Tizard RI Immunity in the fetus and in the newborn Introduction to Veterinary Immunology 2008 8 Barcelona Spain: Elsevier Saunders 223 238

[B11] Berra G Osacar G Mate A Novedades sobre calostro: consideraciones a l ahora de prevenir fallas en el Calostrado Vet Arg 2000 17 282 284

[B12] Carlander D Olesen H Kollberg H Johanesson M Wejaker PE Larsson A Avian antibodies can Eliminate interference due to complement activation in ELISA Upsala J Med Sci 2001 17 282 284 10.3109/2000-1967-145 12166511

[B13] Barroso P Murcia H Vega N Perez G Obtención y purificación de l aIgY dirigidas contra la lectina de Salvia bogotensis Biomedica 2005 25 496 510 16433176

[B14] Chacana PA Terzolo HR Gutiérrez CE Schade R Tecnología IgY o aplicaciones de los anticuerpos de yema de huevo de gallina Rev Med Vet 2004 85 179 189

[B15] Shin JH Yang M Nam SW Use of egg yolk derived immunoglobulin ace an alternative to antibiotic treatment for Control of Helicobacter pylori infection Clin Diagn Lab Immunol 2002 9 1061 1066 12204960 10.1128/CDLI.9.5.1061-1066.2002 PMC120060

[B16] Chalghoumi R Beckers Y Portetelle D Thewis A Hen egg yolk antibodies (IgY), production and use for passive immunization against bacterial enteric infections in chicken Biotech Agron Soc Environ 2009 13 295 308

[B17] Kim MSH Yoshitomo I Han-Chul A Hong-Yon C Hatta H Egg yolk antibody and its application BiotechnolBioprocess Eng 2000 5 79 83

[B18] Huang L Fang X Immunoaffinity Fractionation of Plasma Proteins by Chicken IgY Antibodies Methods Mol Biol 2008 425 41 51 18369885 10.1007/978-1-60327-210-0_4

[B19] Yuzhu Z Jinghui F Huixia F Tanqing L Xiaobo Z Prophylactic and therapeutic effects of egg yolk immunoglobulin against porcine transmissible gastroenteritis virus in piglets Front Agr China 2008 3 104 108 10.1007/s11703-008-0080-9 PMC7088736 32214986

[B20] Gutiérrez CEJ Toledano HM Hans B Rüdiger S Producción de un anticuerpo IgY especifico contra el antígeno CD41 humano Revista CENIC Ciencias Biológicas 2009 40 167 171

[B21] U.S. Department of Health and Human Services National Institutes of Health and the Guide for the Care and Use of Laboratory Animals 2011 8 Washington, DC, Bethesda, MD, USA: National Academy Press

[B22] Murray R Kaplan LA, Pesce AJ Alanine aminotransferase Clinical Chemistry: Theory analysis, and correlation 1989 2 Saint Louis: the CV Mosby company 895 898

[B23] Gendler S Kaplan LA, Pesce AJ **γ-GT** Methods in Clinical Chemistry 1984 Saint Louis, Toronto Princeton: the CV Mosby company 1120 1123

[B24] Burtis CA Ashwood ER Bruns DE Clinical chemistry and molecular diagnostic Tietz textbook of clinical chemistry and molecular diagnostic 2012 5 St Louis Missouri: Elsevier Health Sciences 913 914

[B25] Kliks R Robison JD Myake J Appraisal of four methods for evaluation of colostral immunity of calves J Dairy Sci 1999 82 59

[B26] SAS Procedures guide for staff computers 1999 Cary, NC, USA: SAS Institute, Inc

[B27] Ramaiah SK A: Toxicologist guide to the diagnostic interpretation of hepatic biochemical parameters Food Chem Toxicol 2007 45 1551 1557 17658209 10.1016/j.fct.2007.06.007

[B28] Zvonko S Piršljin J Milinković-Tur S Maja Z-T Ljubić BB Activities of AST, ALT and GGT in clinically healthy dairy cows during lactation and in the dry period Vet Arhiv 2005 75 67 73

[B29] Huang X Yang-KC Hyung-Soon I Oktay EY Hak-Sung K Aspartate aminotransferase (AST/GOT) and alanine aminotransferase (ALT/GPT) Det Tech Sens 2006 6 756 782

[B30] Hsueh CJ Wang JH Dai L Liu CC Determination of alanine aminotransferase with an electrochemical nano ir-C Biosensor for the screening of liver diseases Biosensors 2011 1 107 117 10.3390/bios1030107 PMC4264364 25586923

[B31] Bouda JV Dvorak E Minksova R Dvorak: The activities of GOT, gamma-GT, alkaline phosphatase in blood plasma of cows and their calves fed desde buckets Acta Vet Brno 1980 49 193 198

[B32] Hadorn U Blum JW Effects of colostrum, glucose or water on the first day of life on plasma immunoglobulin G concentrations and gamma-glutamyltransferase activities in calves Zentralbl Veterinarmed A 1997 44 531 537 9465773 10.1111/j.1439-0442.1997.tb01139.x

[B33] Lombardi P Avallone L Pagnini U D'angelo D Bogin E Evaluation of buffalo colostrum quality by estimation of enzyme activity levels J Food Prot 2001 64 1265 1267 11510674 10.4315/0362-028x-64.8.1265

[B34] Campos M Determination of serum activity of the enzyme gamma-glutamyltransferase (γ-GT) as indicator of consumption of colostrum in calves Bachelor Thesis 2000 Valdivia Chile: Universidad Austral de Chile, Facultad de Ciencias Veterinarias, Instituto de Ciencias Veterinaria 1 31

[B35] Singh AK Pandita S Vaidya MM Singh SV Chandra G Pampoori ZA Huozha R Pathan MM Kushwaha R Sharma VK Bovine colostrums and neonate immunity-A Review Agri Review 2011 32 79 90

[B36] Kehoe SI Jayarao BM Heinrichs AJ A survey of bovine colostrum composition and colostrum management practice on Pennsylvania dairy farms J Dairy Sci 2007 90 4108 4116 17699028 10.3168/jds.2007-0040

[B37] Ferraro SS Mendoza GC Marquez AY Marquez AA López-Ortega A Perfil lipídico en becerras mestizas Carora durante el primer año de vida, en época de lluvias y de sequía, en Venezuela http://go.galegroup.com/ps/i.do?id=GALE%7CA197803031&v=2.1&u=pu&it=r&p=IFME&sw=w&asid=cfc42af0c48c5d2a1632b2d678586030

[B38] Matheus N Ramírez F Salazar C Leonardi F Bravo H Relationship albumin: globulin plasma into three times of year in cows of race Carora at different times of the year Gaceta de Ciencias Veterinarias 2001 7 4 10

[B39] Piccione G Casella S Giannetto C Vazzana I Niutta P Giudice E Influence of age on profile of serum proteins in the calf Acta Vet Beograd 2009 59 413 422

[B40] Parish SM Tyler JW Besser TE Gay CC Krtytenberg D Prediction of serum IgG1 concentration in Holstein calves using serum gamma glutamyltransferase activity J Vet Intern Med 1997 11 344 347 9470159 10.1111/j.1939-1676.1997.tb00478.x

[B41] Maden M Birdane FM Altunok V Dere S Serum and colostrums/milk alkaline phosphates activities in the determination of passive transfer status in healthy lambs Revue Med Vet 2004 155 565 569

[B42] Quigley JD Kost CJ Wolfe TM Absorption of protein and IgG in calves fed to colostrum supplement or replacer J Dairy Sci 2002 85 1243 1248 12086061 10.3168/jds.S0022-0302(02)74188-X

[B43] Morrill K Tyler H Two methods to determine IgG concentration in calf serum AS Leaflet 2012 R2708

[B44] Jozica J Nemec M Malovrh T Klinkon M Indicators of passive immunity and health status of calves Acta Vet Beograd 2010 60 513 523

